# Construction of Spatiotemporal Difference Image of the Impact of Financial Structure on China's Total Factor Productivity

**DOI:** 10.1155/2021/7468866

**Published:** 2021-11-24

**Authors:** Yong Yu

**Affiliations:** School of Economics and Finance of Xi'an Jiaotong University, Xi'an 710061, China

## Abstract

My country's current research on the influencing factors of total factor productivity has problems such as single evaluation method, low efficiency, and poor overall level in terms of evaluation methods and evaluation efficiency. Based on this, this study divides the financial structure into three traditional sections, banking, securities, and insurance, and uses the DEA model to study the temporal and spatial differences of the financial structure's influence on the total factor productivity of the four major political and economic regions of China's eastern, western, central, and northeastern China. First, establish a DEA model based on data mining algorithms, combine financial data comparisons over the years, to achieve a quantitative analysis of the financial structure's impact on China's total factor productivity, calculate financial efficiency, and then combine the DEA analysis data model with the grey correlation method. Analyze its internal influence rules, and design experiments for model verification analysis. The results show that the DEA analysis model can realize 8 iterations of data on the impact of financial structure on China's total factor productivity, and its evaluation accuracy can reach more than 96.2%.

## 1. Introduction

In recent years, with the deepening of China's reform and opening up and deepening reform, China's financial structure has also been iteratively optimized, which has been greatly changed and improved. In this process, economic and financial research has played a vital role [1]. In the 21st century, with the emergence and application of the data model, the economic analysis model, and the DEA model, an opportunity for large-scale analysis and calculation of total factor productivity based on informatization and digitization is provided [2]. At present, data diversification and intelligent computing have become the main objectives of the economic and financial research community in the analysis of various economic indicators [3]. Although the existing total factor productivity evaluation system provides a large number of objective evaluation schemes; for example, it puts forward effective schemes from the heterogeneous level [4] and the spatial panel level [5]. However, the analysis of the factors affecting total factor productivity is relatively simple, and for total factor productivity, it often only focuses on one aspect of total factor productivity, such as green total factor productivity [6] or enterprise total factor productivity [7], and there is little research on China's total factor productivity. Most total factor productivity studies are based on the selected targeted evaluation model to achieve the optimal analysis effect [8]. Based on this background, this paper studies the spatial spillover effect of new nanomaterial emerging industry agglomeration on regional economic growth and puts forward a spatial spillover effect evaluation method of new nanomaterial industry regional economic growth based on the DEA model.

In view of the problems of the single evaluation method, low efficiency, and poor overall level in the current research on the influencing factors of total factor productivity in China, this paper studies the spatiotemporal difference image model of the impact of financial structure on China's total factor productivity based on the DEA model and the grey correlation method, which is mainly divided into three parts.  Section 2 mainly introduces the evaluation scope and evaluation methods of different total factor productivities at home and abroad, introduces the research status of total factor productivity data analysis, and points out its research pain points.  Section 3 mainly constructs a regional economic evaluation model of the impact of financial structure on China's total factor productivity based on the DEA model and constructs an information cloud analysis system for total factor productivity. In Section 4, the grey correlation method is used to test the impact of the financial structure based on the DEA model on China's total factor productivity on time and space scales and analyze the results and draw a conclusion.

Compared with the current data description method commonly used on spatiotemporal difference graphs and the a priori algorithm and FP-tree frequency set algorithm commonly used in association analysis, this model can realize 8 iterations of data on the impact of financial structure on China's total factor productivity. And its evaluation accuracy can reach more than 96.2%. This is because the innovation of this paper is to construct the evaluation method of the financial structure's impact on China's total factor productivity through the DEA model. On this basis, not only the model can achieve multiregional evaluation but also the record and storage of the financial structure and China's total factor productivity impact can make full use of the spatial spillover effect information such as the economic expenditure and income record data between each region to calculate the financial efficiency of each financial sector. On the contrary, the grey correlation method is used to quantitatively describe the correlation between the economic behaviors of various regions and regional locations, and the financial structure and China's total factor productivity are prioritized by quantitative indicators. The combination of image recognition methods can effectively affect different regions.

## 2. Related Work

In recent years, there are problems of low efficiency, low reliability, and low data utilization in the evaluation of the influence of financial structure all over the world, especially in the management of financial structure economy in different regions and data analysis of regional economy [9]. As the direction of total factor productivity has always been a hotspot in economic analysis, Zhao et al. used the Porter hypothesis of the second-order least square method to estimate the impact of environmental regulations on the total factor productivity of carbon intensive industries. The results showed that the impact of the two increased first and then decreased, indicating that, under the influence of the previous subsidy policy, carbon intensive industries are gradually developing towards self-discipline and compliance [10]. Zhang et al. calculated the green total factor productivity of China's food industry by the DEA Malmquist method. According to the calculation results, researchers are optimistic about the technical efficiency of China's food industry [11]. Haider and Bhat, in India, analyzed the relationship between energy efficiency and total factor productivity through the GMM-IV model for the paper industry in 21 Indian states. The research found that when the energy efficiency consumed per unit output decreases, the total factor productivity will increase. Therefore, it is urgent to develop energy-saving technology and reduce the heterogeneity of industrial structure [12]. Houedjofonon et al. have studied the relationship between family farm economy and total factor productivity in Benin, Africa. Through the study of the translog function, it is found that the impact of family farm economy on productivity is negative, and the government can encourage the development of farm economy [13]. Haller and Lyons studied the impact of the popularization of broadband network in Ireland on the total factor productivity of enterprises through the spatial information correlation method and found that compared with the service industry, the popularization of network has a greater impact on the total factor productivity of administrative support enterprises [14]. Lin and Ge used the SBM method, the Malmquist–Luenberger index, and the DEA model to analyze China's forest ecological economic efficiency and forest total factor productivity and found 8 characteristics that have an impact on forest total factor productivity. These characteristics may have guiding opinions on the introduction of bills and policies related to forest protection [15]. Rodriguez et al. analyzed the dynamic evolution of total factor productivity of wind farms in Spain based on the Malmquist index. The index combines the two analysis evaluation models for analysis, so as to evaluate the impact of different technologies used in the wind farm. The results show that almost all the weak growth of total factor productivity comes from the growth of technical productivity, and technological change does not contribute to this. It shows that the technical capacity of wind farms in Spain has not been improved for many years [16]. Lanz et al. analyzed the dynamic evolution of agricultural total factor productivity by introducing random factors. The research and analysis results show that agricultural total factor productivity has a profound impact on population and consumption. In order to prevent temporary production shortage, the land structure should convert more land into agricultural land [17]. Aiming at the renewable energy efficiency and total factor productivity of the European Union, Gökgöz and Güvercin used the super DEA model and the Malmquist–Luenberger index analysis to find that technological innovation and technology diffusion are the driving source of the total factor growth rate of renewable energy. This research provides theoretical support for the formulation of energy policies in various countries [18]. Similarly, Sugathan et al. improved the traditional total factor productivity by using the stochastic metafrontier model, evaluated the operation performance and total factor productivity of coal and natural gas power plants at the time level, and found that the structural reform had no positive impact on the natural gas power plant, but on the technological frontier, production efficiency and production scale were improved [19]. Aiming at China's metal industry, Feng et al. analyzed the provincial green total factor yield in China through the metafrontier model and found that the main reasons for the low green total factor yield in the metal industry are relatively backward technical efficiency and obvious spatial distribution [20].

Based on the above research status at home and abroad, it can be seen that the current research on total factor productivity at home and abroad is mainly focused on energy, agriculture, industry, or environment [21–23]. And most studies do not involve the cloud data analysis and evaluation model of the impact of financial structure on China's total factor productivity. Furthermore, we do not pay attention to the research results of constructing and characterizing the data in the form of spatiotemporal difference images. Therefore, it is of great significance to study the spatiotemporal difference image construction of the impact of financial structure based on the DEA model on China's total factor productivity.

## 3. Methodology

### 3.1. Application of the DEA Model in the Evaluation System of the Impact of Financial Structure on China's Total Factor Productivity

The algorithms were used in traditional financial data analysis models (such as a priori algorithm and FP-tree frequency set algorithm); in the process of analyzing financial data, the data dimension of a single analysis can only be set before the analysis. And it is impossible to adaptively determine the number of analysis according to the types of different data groups, so the final analysis result has a direct impact on the artificially set single maximum analysis dimension value [24]. The DEA model is a model that can evaluate the effective efficiency of multiple decision-making units with *n* input or *n* output attributes. Compared with other financial research models, the DEA model has the advantage of not needing to calculate the standard cost of each subproject, that is, there is no need to calculate the standard cost of each subproject. The input and output content is converted into the same financial unit, but *n* output and *n* input are directly converted into the denominator and numerator for calculating the efficiency ratio [25]. Therefore, when the DEA model is used to calculate the efficiency of the financial structure, the input and output components can be obtained simply and clearly. Therefore, compared with the traditional market share or profit value, the DEA model has high calculation reliability, strong evaluation ability, and other characteristics. The evaluation method based on DBA can calculate all financial structure outputs and all financial structure inputs of the impact of financial structure on China's total factor productivity through the denominator and the numerator of the efficiency ratio without calculating the standard cost of all factors affected by financial structure on China's total factor productivity. This calculation method can calculate the unit involved in the problem that does not involve the impact of the traditional financial structure on China's total factor productivity. Therefore, in the evaluation and calculation of the impact of financial structure on China's total factor productivity, in order to realize the financial structure between different regions, the economic data can quickly construct the spatiotemporal difference image and store it in the big data information system for analysis. This study records the economic data of financial structure with time and regional differences in real time through the integrated big data system and analyzes the impact of financial structure on China's total factor productivity by using the data envelopment analysis model (DEA) and double coupling factors. This study divides the traditional financial structure into three, selects banking, insurance, and industry as the characteristics, constructs a management system of the impact of financial structure on China's total factor productivity based on Hadoop framework, and constructs the spatiotemporal difference image according to the processed data. The construction of spatiotemporal image has theoretical support and guiding significance for optimizing the financial structure and realizing the hierarchical framework of the impact evaluation system of financial structure on China's total factor productivity and is of great practical significance to regional financial management departments.

**Figure 1:**
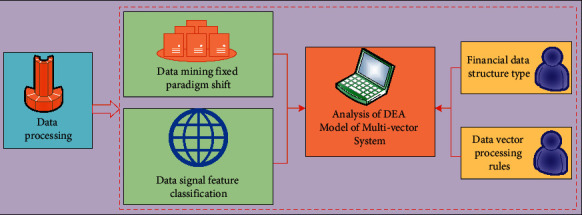
Data processing flow of the DEA model of multivector system.

On the contrary, if we want to implement the model based on data mining, we have to convert the mined data into a signal that can be discriminated by the program through some fixed paradigm. Relevant researchers generally carry out vector spatial processing on the financial structure data in different regions to overcome this problem. In this way, the change of the financial structure can be analyzed and evaluated from multiple angles. For the DEA model of multivector system, the data processing flow is shown in Figure 1.

### 3.2. The Analytic Process of the DEA Model in the Evaluation System of Financial Structure on China's Total Factor Productivity

Total factor productivity is defined as the residual in the economic growth rate other than the contribution of capital and labor. In order to analyze China's total factor productivity, this study divides China into four plates, east, west, central, and northeast, according to political and economic regions, and divides the economic structure into three plates, banking, insurance, and securities. Firstly, this study assumes that there is a dependency between financial structure and total factor productivity. Then, China's total production factor *Q* can be expressed as the following formula:

(1) $$\begin{array}{c} Q\left({\text{in}}_{it},{\text{some}}_{it}\right)=Q_{i0}\times {\text{in}}_{it}^{x_{1}}\times {\text{some}}_{it}^{x}, \end{array}$$

where *i* represents a certain region, *t* represents a certain time, in_*it*_ represents the efficiency index of a certain financial sector, some_*it*_ represents the control variable, and *x* and *x*_1_ are the influence parameters, respectively. Then, the total production value *Z* in domain *i* can be expressed as the following formula:

(2) $$\begin{array}{c} Z_{it}=Q_{it}\times f\left(L_{it},K_{it}\right), \end{array}$$

where *f* represents function and *L* and *K* represent labor input and capital input, respectively. Combine formulas (1) and (2) to obtain

(3) $$\begin{array}{c} Z_{it}=Q_{i0}\times f\left(L_{it},K_{it}\right)\times {\text{in}}_{it}^{x_{1}}\times {\text{some}}_{it}^{x}. \end{array}$$

In combination with equation (3), the productivity index is decomposed by the DEA model to obtain

(4) $$\begin{array}{c} Q_{it}=\frac{Z_{it}}{f\left(L_{it},K_{it}\right)},\\ \frac{Z_{it}}{f\left(L_{it},K_{it}\right)}=Q_{i0}\times {\text{in}}_{it}^{x_{1}}\times {\text{some}}_{it}^{x}. \end{array}$$

After transformation, the above results are obtained:

(5) $$\begin{array}{c} \ln\;Q_{it}=\ln\;Q_{i0}\times x_{1}\ln\;{\text{in}}_{it}\times x\;\ln\;{\text{some}}_{it}. \end{array}$$

In this study, the impact of financial structure on total factor productivity is divided into banking element *R*, insurance element *S*, and securities element *T*. Due to the dynamic characteristics of the financial structure, this study adds personality difference *V* and random interference *μ* into the model constructed by equation (5) and obtains

(6) $$\begin{array}{c} \ln\;Q_{it}=\alpha +\alpha_{0}\ln\;Q_{it-1}+\alpha_{1}\ln\;{\text{in}}_{it}+\eta_{1}^{\prime }x\;\ln\;{\text{some}}_{it}+V_{1i}+\mu_{1it},\\ \ln\;R_{it}=\beta +\beta_{0}\ln\;R_{it-1}+\beta_{1}\ln\;{\text{in}}_{it}+\eta_{2}^{\prime }x\;\ln\;{\text{some}}_{it}+V_{2i}+\mu_{2it},\\ \ln\;S_{it}=\chi +\chi_{0}\ln\;S_{it-1}+\chi_{1}\ln\;{\text{in}}_{it}+\eta_{3}^{\prime }x\;\ln\;{\text{some}}_{it}+V_{3i}+\mu_{3it},\\ \ln\;T_{it}=\delta +\delta_{0}\ln\;T_{it-1}+\delta_{1}\ln\;{\text{in}}_{it}+\eta_{4}^{\prime }x\;\ln\;{\text{some}}_{it}+V_{4i}+\mu_{4it}. \end{array}$$

*α*, *β*, *χ*, and *δ* are all undetermined parameters, and *η* is a set of undetermined parameters. Based on the above analysis, this study preliminarily establishes a model of the impact of financial structure on China's total factor productivity based on the DEA method.

### 3.3. A Confirmatory Experimental Design of the DEA Model on China's Total Factor Productivity in Financial Structure

**Figure 2:**
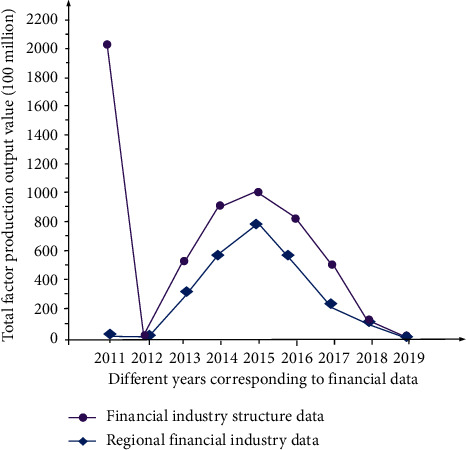
Corresponding financial industry data and structural data changes from 2011 to 2019.

After establishing the DEA model, it is necessary to discuss the analysis process and effectiveness of total factor productivity in the financial structure. Therefore, the first step is to build a data base. In this study, the data of three financial structures of banking, insurance, and securities in the four regions of eastern, western, central, and northeastern China from 2011 to 2019 are mined as samples, and the banking element *R*, insurance element *T*, and securities element *S* affecting total factor productivity are collectively referred to as financial efficiency. Calculate the financial efficiency of each financial industry, and the calculation results are shown in Table 1:

The coupling statistical analysis results of financial industry data in different regions and overall are shown in Figure 2.

According to the calculation results in Table 1 and Figure 2, from 2011 to 2019, the financial structure was gradually optimized and showed a change trend of increasing first and then decreasing, which is directly related to the government's policies and the reform of the financial industry as a whole (Internet finance and blockchain finance). Overall, the former banking industry has become the mainstream of the financial structure, and the insurance and security industry has a trend of spreading from the previous exclusive gathering in the eastern region to the central, western, and northeast regions. From the perspective of microregions, the northeast region will be evenly distributed in the structure of banking, insurance and securities industries until 2019, while the eastern region will have an absolutely dominant financial structure distribution of security industry before 2015. Towards 2019, the security industry in the western region will gradually become the main financial structure, while the proportion of security financial structure in the central region will gradually decline. On the contrary, the financial structure of its security industry accounts for the latter.

## 4. Result Analysis and Discussion

### 4.1. Construction of the DEA Model in the Spatiotemporal Difference Image of the Impact of Financial Structure on China's Total Factor Productivity

**Figure 3:**
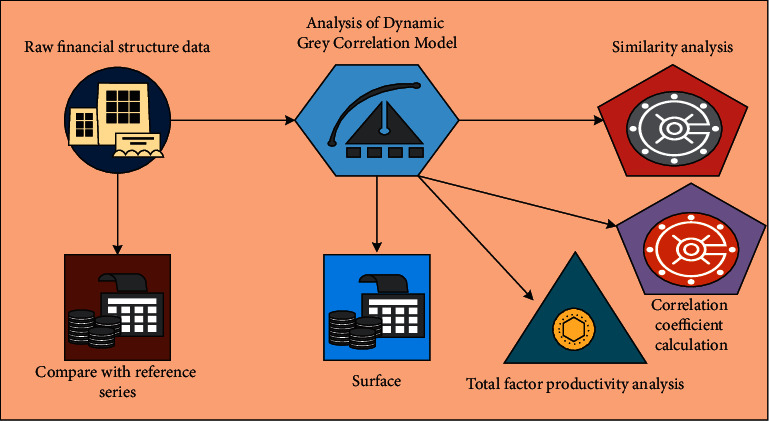
The analysis process of the dynamic grey relational model on financial data and total factor production data.

In order to better explore the impact of financial structure on China's total factor productivity, this study calculates the correlation degree between financial efficiency and total factor productivity through the method of the grey correlation model. Its basic idea is to explore the geometric similarity between comparison sequence and reference sequence. The traditional association coupling model is weak in dealing with complex discrete dynamic data. This study combines with data mining technology to give the dynamics that the traditional association coupling model does not have, so as to effectively construct spatiotemporal difference images. The basic steps are as follows: firstly, take the relevant data of financial efficiency of each financial structure as the comparison sequence and the relevant data of China's total factor productivity as the reference sequence, then calculate the correlation coefficient between them, and, finally, calculate the correlation degree based on the correlation coefficient.  Figure 3 shows the flowchart of the calculation method.

Next, the construction of the dynamic grey correlation model will be specifically analyzed. In this study, *α* is defined as the correlation coefficient and Ψ as the correlation degree. In order to calculate them, *u* needs to be defined as the mean image of each sequence, *U* as the matrix of the mean image, Δ as the matrix of the difference sequence, and *m* and *M* as the minimum difference and maximum difference of the two poles. The calculation formulas of *α* and Ψ are as follows. First, calculate the initial mean image of each sequence:

(7) $$\begin{array}{c} U_{i}^{\prime }=\frac{U_{i}}{x_{i}\left(1\right)}=\left[u_{1}^{\prime }\left(1\right),u_{1}^{\prime }\left(2\right),\ldots ,u_{i}^{\prime }\left(n\right)\right],\;i=0,1,2,\ldots ,m. \end{array}$$

Then, calculate the difference sequence:

(8) $$\begin{array}{c} \Delta_{i}\left(k\right)=\left|u_{0}^{\prime }\left(k\right)-u_{i}^{\prime }\left(k\right)\right|,\\ \Delta_{i}=\left[\Delta_{i}\left(1\right),\Delta_{i}\left(2\right),\ldots ,\Delta_{i}\left(n\right)\right]\text{,}\\ i=0,1,2,\ldots ,m. \end{array}$$

Minimum and maximum difference between the following two poles:

(9) $$\begin{array}{c} M=\underset{i}{\max}\underset{k}{\max}\Delta_{i}\left(k\right),\\ m=\underset{i}{\min}\underset{k}{\min}\Delta_{i}\left(k\right). \end{array}$$

Based on the above calculation, the correlation coefficient *α* is obtained:

(10) $$\begin{array}{c} \alpha_{0i}\left(k\right)=\frac{m+\beta M}{\Delta_{i}\left(k\right)+\beta M},\;k=1,2,\ldots ,n;\;i=1,2,\ldots ,n, \end{array}$$

where *β* is the coefficient in the range of 0 to 1. Finally, the judgment coefficient Ψ can be obtained:

(11) $$\begin{array}{c} \Psi_{0i}=\frac{1}{n}\sum_{k=1}^{n}\alpha_{0i}\left(k\right),\;k=1,2,\ldots ,n;\;i=1,2,\ldots ,n. \end{array}$$

**Figure 4:**
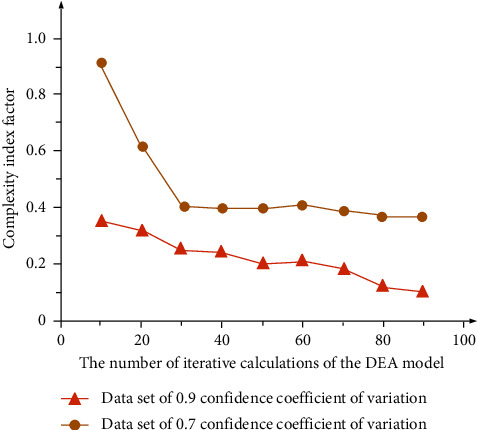
DEA model simulation analysis results of financial data under different confidence variation coefficients.

The evaluation coefficient is an index for quantitative evaluation of the internal correlation between different data according to the association rule of the Gaussian normal distribution function. Generally, when Ψ is greater than 0.8, the reference attribute and contrast attribute are strongly correlated, when Ψ is less than 0.8 and greater than 0.5, they are moderately correlated, when Ψ is less than 0.5 and greater than 0.3, they are weakly correlated, and when Ψ is less than 0.3, they are poorly or even not correlated. According to the calculated correlation coefficient, the correlation degree between the comparison sequence and the reference sequence can be obtained. For the convenience of calculation, this study takes the financial efficiency of banking, insurance, and securities as the comprehensive financial structure efficiency, takes the comprehensive financial structure efficiency as the comparison sequence, and takes the total factor of production efficiency as the reference sequence. The simulation analysis results of three groups of known data are shown in Figure 3. After the calculation, it is necessary to simulate and analyze the data of the impact of financial structure on China's total factor productivity. The simulation analysis results are shown in Figure 4.

As can be seen from Figure 4, in the analysis results of two groups of financial structure data groups with different correlation degrees (set the confidence change coefficient of 0.7/0.9, respectively), the complexity index factor of the DEA model changes with the number of calculations, and its change trend shows a gradual downward trend. When the confidence change coefficient is 0.9, the complexity index factor is basically in a stable state (maintained below 0.4). The main reason for this trend is that the new scheme combining grey correlation algorithm with the DEA model not only inherits the previous function of intelligently screening the correlation information of the impact of financial structure on China's total factor productivity but also can be self-optimized according to dynamic data import.

### 4.2. Experimental Results and Analysis

**Figure 5:**
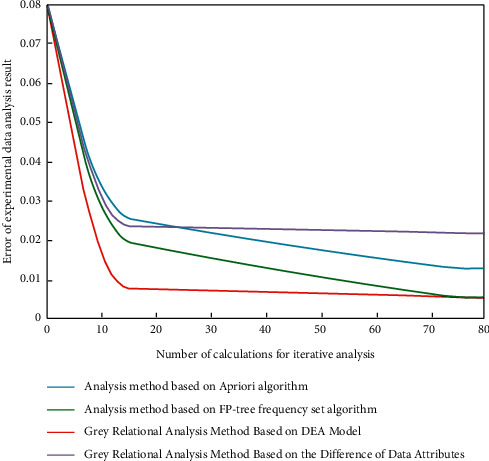
Experimental simulation analysis results of two mainstream data analysis strategies and grey management degree.

This study selects China's total factor productivity data from 2011 to 2019 calculated by the formula in Section 3 as the reference sequence of changes in total factor productivity data and selects the comprehensive financial structure efficiency in these 9 years as the comparison sequence of changes in financial structure, so as to better first construct the spatiotemporal difference image of the impact of financial structure on China's total factor productivity. In order to determine the excellent performance of the grey correlation method based on the DEA model, this study analyzes and compares the comparative experiments of the mainstream a priori algorithm, FP-tree frequency set algorithm, and the grey correlation analysis strategy based on data attribute differences. The results are shown in Figure 5.

**Figure 6:**
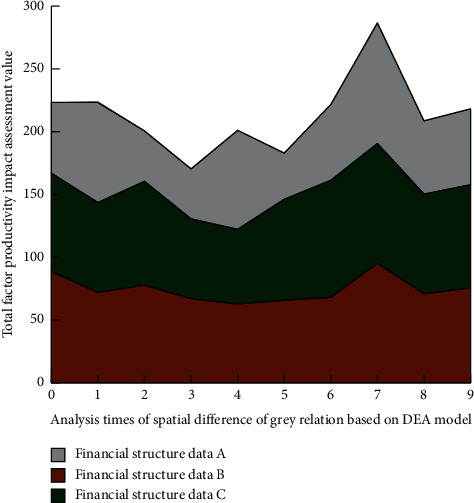
Spatial difference map of the impact of financial structure on China's total factor productivity in 2013.

It can be seen from Figure 5 that, among the four methods, compared with the other three mainstream methods (a priori algorithm, FP-tree frequency set algorithm, and grey correlation analysis strategy based on data attribute difference), the grey correlation method based on the DEA model has smaller error degree with the increase of iterative analysis times because the DEA model is in the process of data analysis. It can realize different levels of analysis and quantitative evaluation according to the correlation degree between data. The obtained data are imported into the big data integration system based on the dynamic grey correlation model designed in this study one by one according to the year, and then, the output results are visualized. In the process of analyzing the experimental results, combined with the correlation analysis characteristics of China's financial structure and China's total factor productivity from 2011 to 2019, this study selects four representative years, 2013, 2015, 2017, and 2019, as the time scale experimental objects. The spatial difference image of the impact of financial structure on China's total factor productivity in 2013 is shown in Figure 6, in which the color bar represents the correlation degree, and A/B/C, respectively, represent the quantitative financial data in the three major sectors of traditional banking, securities, and insurance.

**Figure 7:**
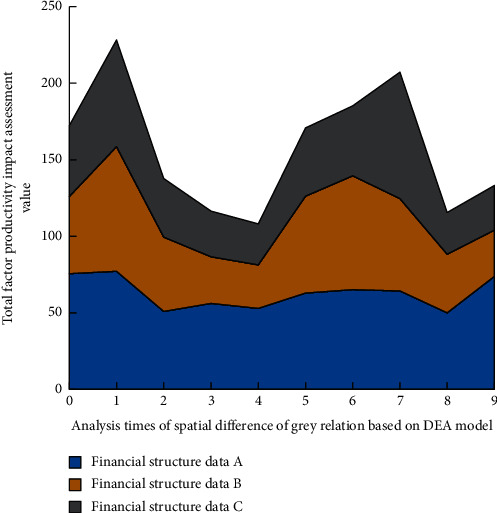
Spatial difference map of the impact of financial structure on China's total factor productivity in 2015.

The spatial difference image of the impact of financial structure on China's total factor productivity in 2015 is shown in Figure 7.

**Figure 8:**
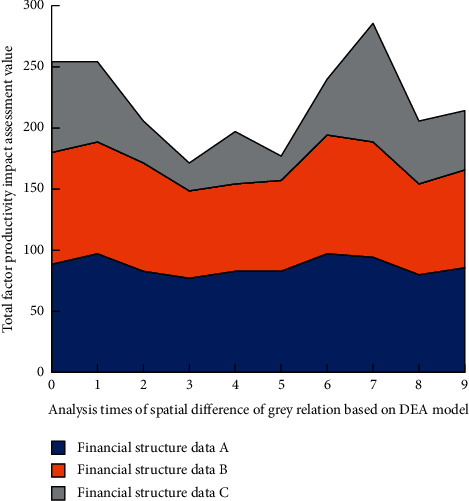
Spatial difference map of the impact of financial structure on China's total factor productivity in 2017.

The spatial difference image of the impact of financial structure on China's total factor productivity in 2017 is shown in Figure 8.

**Figure 9:**
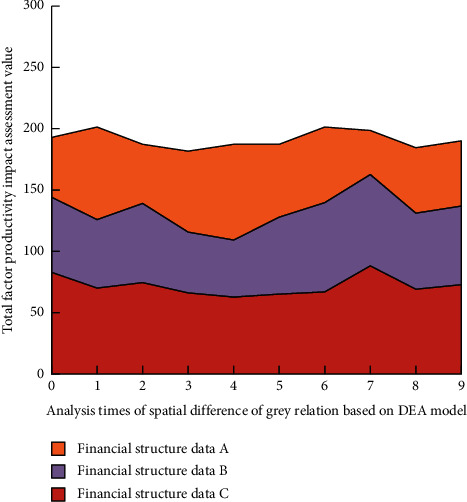
Spatial difference map of the impact of financial structure on China's total factor productivity in 2019.

The spatial difference image of the impact of financial structure on China's total factor productivity in 2019 is shown in Figure 9.

Through the analysis of Figures 6∼9, it can be found that, in local space, with the increase of the number of spatial difference analysis of grey correlation based on the DEA model, among the three different financial data groups, the impact of financial structure on China's total factor productivity is generally medium. However, in eastern China, the financial structure has a great impact on China's total factor productivity, and its position is difficult to shake. Before 2017, the impact of the financial structure in Western China and Northeast China on China's total factor productivity was low. After 2017, the impact of the financial structure in Western China on China's total factor productivity came from behind. At the same time, the impact in Northeast China changed little. In the overall space, the high correlation of the impact of financial structure on China's total factor productivity shows a diffusion trend centered on the East on the spatial scale. The above results show that China's financial structure is gradually diversified and healthy. The graphical results roughly reflect the impact of China's financial structure on China's total factor productivity and have certain guidance for China's economic work.

## 5. Conclusion

There are some problems in the evaluation of the impact of China's current financial structure on China's total factor productivity, such as single evaluation method, low evaluation efficiency, and poor overall evaluation level. The analysis results of traditional financial data analysis models (such as the a priori algorithm and the FP-tree frequency set algorithm) have a direct impact on the artificially set single maximum analysis dimension value, and the DEA model has high calculation reliability and strong evaluation capabilities. The advantage is that there is no need to calculate the standard cost of each subproject; that is, there is no need to convert the input and output content into the same financial unit, but directly convert the multidimensional output and multidimensional input into the denominator, and the denominator is used to calculate the efficiency ratio. The molecule can realize adaptive analysis. Based on this, this paper studies the spatiotemporal difference image construction of the impact of financial structure on China's total factor productivity based on the DEA model and grey correlation analysis. Firstly, the DEA financial structure analysis model based on data mining technology is established to realize the data storage and analysis in the process of financial structure analysis and summarize the temporal and spatial differences of the financial structure. Then, combined with the data of China's total factor productivity over the years, the results are fed back to the DEA model for correlation analysis, and relevant experiments are designed to verify the model. The experimental results show that compared with other analysis methods, this model can analyze the multilayer data of the financial structure, which greatly improves the evaluation efficiency and objectivity. The analyzed model uses image processing technology for data reprocessing, which can give a visual analysis of the experimental results from the perspective of the data. It is not necessary to set the single maximum analysis dimension before analyzing the data, but can be based on the actual dimensional characteristics of the data group. To achieve its adaptive analysis, the efficiency and accuracy of analysis are improved, which provides a way for researchers to analyze massive, multidimensional financial data or predict the effect of future financial structure (data type and dimension uncertain). This new method can also be used in the future to combine the characteristics of the financial structure of different industries to achieve in-depth research on variable weight analysis.

## Figures and Tables

**Table 1:** Financial efficiency of banking, insurance, and security industry in east, west, central, and northeast China from 2011 to 2019.

Regional/financial structure	2011	2012	2013	2014	2015	2016	2017	2018	2019
East China/banking	0.312	0.333	0.281	0.251	0.221	0.189	0.176	0.137	0.124
East China/insurance industry	0.321	0.365	0.412	0.452	0.423	0.512	0.554	0.610	0.711
East China/security business	0.531	0.541	0.635	0.778	0.871	0.980	0.941	0.991	0.987
West China/banking	0.453	0.462	0.450	0.411	0.397	0.343	0.323	0.299	0.300
West China/insurance industry	0.394	0.396	0.427	0.461	0.514	0.558	0.560	0.577	0.601
West China/security business	0.522	0.512	0.621	0.622	0.631	0.678	0.781	0.711	0.824
Central China/banking	0.523	0.514	0.422	0.494	0.441	0.384	0.379	0.276	0.311
Central China/insurance industry	0.481	0.501	0.511	0.666	0.621	0.707	0.712	0.782	0.771
Central China/security business	0.212	0.247	0.233	0.343	0.358	0.375	0.388	0.451	0.411
Northeast China/banking	0.583	0.574	0.527	0.491	0.501	0.41	0.328	0.331	0.357
Northeast China/insurance industry	0.228	0.275	0.266	0.280	0.314	0.337	0.382	0.364	0.351
Northeast China/security business	0.124	0.214	0.258	0.314	0.387	0.401	0.365	0.322	0.325

## Data Availability

The experimental data used to support the findings of this study are available from the corresponding author upon request.
